# Pediatric dentistry procedures performed within the Brazilian National Health System in the state of Rio Grande do Sul before and during the COVID-19 pandemic: difference between the years 2018 and 2021

**DOI:** 10.1590/S2237-96222023000100008

**Published:** 2023-03-03

**Authors:** Hellen Monique da Motta, Lara Emmile Evangelista Valença, Luiza Helena de Souza Fernandes, Rafaela Corrêa Martins, Letícia Regina Morello Sartori, Sarah Arangurem Karam

**Affiliations:** 1 Universidade Federal de Pelotas, Faculdade de Odontologia, Pelotas, RS, Brazil; 2 Universidade Federal de Pelotas, Programa de Pós-Graduação em Odontologia, Pelotas, RS, Brazil

**Keywords:** Epidemiology, Descriptive, Tooth, Deciduous, Dental Care, COVID-19, Brazilian National Health System, Ecological Studies

## Abstract

**Objective:**

to analyze the difference in the number of primary teeth dental procedures performed within the Brazilian National Health System (SUS) in the state of Rio Grande do Sul, before and during the COVID-19 pandemic.

**Methods:**

this was a descriptive ecological study, using secondary data from the SUS Outpatient Information System (SIA-SUS), from 2018 to 2021, in the state and in its seven health macro-regions; we calculated the relative and absolute frequencies and the percentage difference of the dental procedures performed.

**Results:**

94,443 and 36,151 dental procedures were recorded before and during the pandemic, respectively, corresponding to a 61.7% reduction; relevant percentage reductions were found in restorative procedures, which reached 20% in the southern region of the state; an increase in the percentage of exodontic and endodontic procedures was found.

**Conclusion:**

the results suggest that the COVID-19 pandemic had negative repercussions on the performance of primary teeth dental procedures in Ro Grande do Sul.

**Study contributions**

**Main results**

There was a reduction in the number of pediatric dental care services provided within the SUS during the pandemic period analyzed (January-June, 2020 and 2021), when compared to the same months of the years prior to the pandemic (2019 and 2018).

**Implications for services**

Following the restriction of care services and suspension of actions to promote children’s oral health, there is a need for specific planning to normalize pediatric dental care within the SUS.

**Perspectives**

Further studies are recommended to monitor pediatric dental care within the SUS in the post-pandemic period and assess shortcomings. The impact on children’s oral health that the reduction in services during the pandemic period may have caused needs to be quantified.

## INTRODUCTION

The Brazilian National Health System (*Sistema Único de Saúde* - SUS) provides free dental care at different levels of complexity, covering various specialties and procedures, including procedures related to pediatric dental care.^1^ Considering the scenario imposed by coronavirus disease (COVID-19) in Brazil, especially during the years 2020 and 2021, and the need to implement restrictive measures regarding interpersonal physical contact,^2^ most dental surgeries only performed emergency treatment and postponed elective dental treatment.^3^ From this perspective, a study carried out with SUS data showed a relevant reduction in the number of general dental appointments in Brazil, ranging from 55%, in the first month of the pandemic, to more than 88% in the following months of 2020.^4^

The COVID-19 pandemic may have substantially impacted children’s oral health. As a result of the restrictive measures, face-to-face school classes were suspended,^5^ this being a factor that prevented the implementation of child oral health promotion and prevention actions in the school environment.^6^ In 2014, 80.9% of SUS oral health teams reported providing care to children up to 5 years old.^7^ More recently, a study showed that Southern Brazil is the region of the country in which children most have dental appointments.^8^

The objective of this study was to analyze the difference in the number of primary teeth dental procedures performed within the Brazilian National Health System in the state of Rio Grande do Sul, before and during the COVID-19 pandemic.

## METHODS

This study has a descriptive ecological design and used secondary data related to dental outpatient production in the state of Rio Grande do Sul (RS), retrieved from the SUS Department of Information Technology (DATASUS) Outpatient Information System (*Sistema de Informações Ambulatoriais* - SIA/SUS) (http://datasus.saude.gov.br). Information on dental procedures performed in RS and in its seven health macro-regions can be obtained from the DATASUS website, from the item “health information” (“*informações de saúde*”), sub-item “outpatient production” (“*produção ambulatorial*”), tabulated using the TabWin software, version 3.52. These data are recorded monthly for the 497 municipalities that make up the state.^9^ ^,^ ^10^ ^,^ ^11^ All data were collected and exported from the online platform in October 2021 by four previously trained reviewers.

We collected absolute data from the DATASUS platform regarding deciduous teeth dental procedures performed, considering the following dental procedure codes: (i) deciduous tooth restoration (0307010023); (ii) posterior deciduous tooth restoration with composite resin (0307010082); (iii) posterior deciduous tooth restoration with amalgam (0307010090); (iv) posterior deciduous tooth restoration with glass ionomer (0307010104); (v) anterior deciduous tooth restoration with composite resin (0307010112); (vi) endodontic treatment of deciduous teeth (0307020037); and (vii) deciduous tooth extraction (0414020120).^12^ ^,^ ^13^

For the purpose of time standardization, the study period was defined as the first semester (January 1st to June 30th) of the years 2018, 2019, 2020 and 2021, since information was incomplete for the year 2021 at the time of data extraction. Data on the absolute number of procedures was collected using procedure codes and stratification by year and place where the procedure was performed (health macro-regions/RS), as per specific fields of the DATASUS platform.

The “type of dental procedure” variable was defined according to the codes initially collected, and categorized as “restorative procedure” (0307010023, 0307010082, 0307010090, 0307010104 and 0307010112), “endodontic procedure” (0307020037) and “tooth extraction” (0414020120).

We categorized the year of dental care in the period 2018 to 2021, as the pre-pandemic period (2018-2019), and the pandemic period (2020-2021). Additionally, the Rio Grande do Sul “health macro-regions” variable was categorized according to the state’s seven health macro-regions: *Vales*, Southern, *Serra*, Northern, *Missioneira*, Metropolitan and Midwest.^14^

The data were exported and organized in a database using Microsoft Excel 2016 software (Microsoft, Redmond, Washington, USA). Descriptive analysis was performed using the same software, considering the absolute and relative numbers of the “type of dental procedure” variable, according to period and health macro-region. Subsequently, we calculated the percentage difference in the number of dental procedures performed between the periods studied, for each type of procedure.

Approval by a Research Ethics Committee was not required for this study since it used anonymous public access secondary data.

## RESULTS

**Figure 1:**
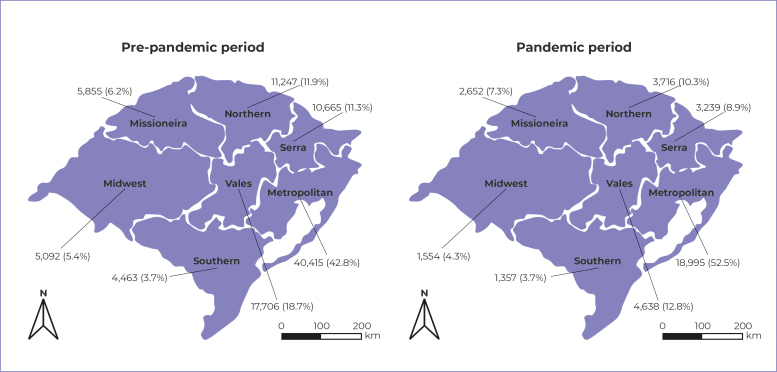
- Total deciduous teeth dental procedures and respective percentages, in the pre-pandemic period (2018-2019) and the pandemic period (2020-2021), by Rio Grande do Sul health macro-regions, 2018-2021

In the period evaluated, a total of 130,594 procedures were performed, i.e. 94,443 in the pre-pandemic period and 36,151 during the pandemic. There was a reduction in the absolute and relative number of deciduous teeth dental procedures performed in all health macro-regions (Figure 1) and in the state of Rio Grande do Sul as a whole (Table 1).

**Table 1:** - Absolute frequencies (n) and relative frequencies (%) of type of deciduous teeth dental procedures and total procedures during the pre-pandemic period (2018-2019) and the pandemic period (2020-2021), Rio Grande do Sul and its health macro-regions, Brazil, 2018-2021

Health macro-region/ Rio Grande do Sul	Pre-pandemic period n (%)			
	Restorative procedure	Endodontic procedure	Extraction procedure	Total procedures
Vales Region	12,240 (69.1%)	421 (2.4%)	5,045 (28.5%)	17,706 (18.7%)
Southern Region	1,976 (57.1%)	237 (6.8%)	1,250 (36.1%)	3,463 (3.7%)
Serra Region	7,280 (68.3%)	78 (0.7%)	3,307 (31.0%)	10,665 (11.3%)
Northern Region	7,872 (69.9%)	87 (0.8%)	3,288 (29.2%)	11,247 (11.9%)
Missioneira Region	3,912 (66.8%)	33 (0.6%)	1,910 (32.6%)	5,855 (6.2%)
Metropolitan Region	22,391 (55.4%)	6,363 (15.7%)	11,661 (28.8%)	40,415 (42.8%)
Midwest Region	3,428 (67.3%)	59 (1.2%)	1.605 (31.5%)	5,092 (5.4%)
Rio Grande do Sul	59,099 (62.6%)	7,278 (7.7%)	28,066 (29.7%)	94,443 (100%)
**Health macro-region/ Rio Grande do Sul**	**Pandemic period n (%)**			
Vales Region	2,524 (54.4%)	220 (4.7%)	1,894 (40.8%)	4,638 (12.8%)
Southern Region	504 (37.1%)	25 (1.8%)	828 (61.0%)	1,357 (3.7%)
Serra Region	1,973 (60.9%)	38 (1.2%)	1,228 (37.9%)	3,239 (8.9%)
Northern Region	2,325 (62.6%)	89 (2.4%)	1,302 (35.0%)	3,716 (10.3%)
Missioneira Region	1,607 (60.6%)	15 (0.6%)	1,030 (38.8%)	2,652 (7.3%)
Metropolitan Region	7,840 (41.3%)	6,050 (31.8%)	5,105 (26.8%)	18,995 (52.5%)
Midwest Region	871 (56.0%)	40 (2.6%)	643 (41.4%)	1,554 (4.3%)
Rio Grande do Sul	17,644 (48.8%)	6,477 (17.9%)	12,030 (33.3%)	36,151(100%)

Table 1 shows the frequency of the types of procedure per region of the state, during the pre-pandemic and pandemic periods. In both periods, the Metropolitan region performed the largest number of procedures, regardless of type. In the pre-pandemic period, restorative procedures were the most prevalent in all regions of the state. Considering the pandemic period alone, the Southern, *Missioneira* and Midwest regions recorded the lowest numbers for all three types of procedure evaluated. Still in the pandemic period, restorative procedures were the most prevalent type of deciduous teeth procedure in almost all regions, except the southern region of the state, where extraction was the most frequent procedure (Table 1).

**Figure 2:**
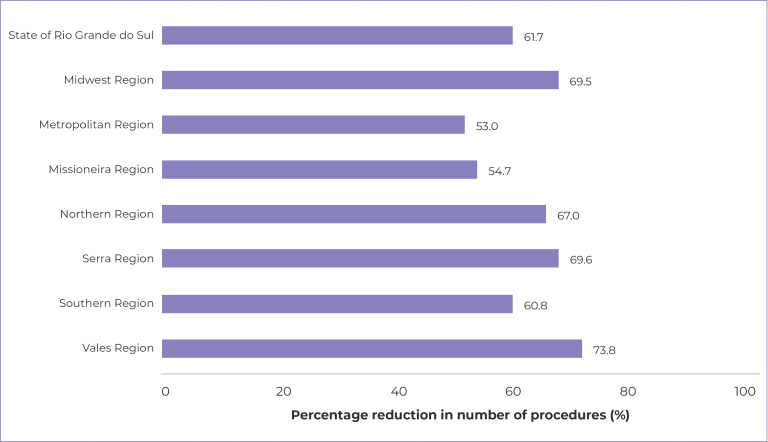
- Percentage reduction in the number of deciduous teeth dental procedures in the pre-pandemic period (2018-2019) and the pandemic period (2020-2021), in Rio Grande do Sul and its health macro-regions, 2018-2021

When comparing the proportions of procedures performed in the two periods assessed, as illustrated in Figure 2, a decrease of more than 50% was observed in all regions analyzed. There was a 61.7% reduction in the state as a whole, with the *Vales* region standing out as having the largest percentage reduction. Moreover, when considering the types of procedures performed during the two periods (2018-2019 and 2020-2021), a 20% reduction was identified in restorative procedures in the Southern region (57.1% *versus* 37.1%, respectively) and a 14.7% reduction in the *Vales* region (69.1% *versus* 54.4%, respectively) (Table 1).

## DISCUSSION

This study identified a reduction of approximately 62% in the performance of deciduous teeth curative dental procedures, in the state of Rio Grande do Sul, during the first semesters of 2020 and 2021, in comparison with the same months in 2018 and 2019. With regard to types of procedure, restorative procedures stood out as having the largest percentage reduction among all regions of the state.

The findings of this study are in line with national data related to children’s dental care during the pandemic.^4^ ^,^ ^5^

The considerable reduction in the number of dental procedures performed may result in a lack of curative/preventive treatment for deciduous teeth.^15^ Failure to perform minimally invasive or restorative procedures may contribute to the subsequent evolution of dental caries lesions, generating dental pain, functional and esthetic alterations that, in turn, may lead to children having poorer quality of life and caregivers facing increased stress.^16^

According to Ministry of Health recommendations,^17^ ^,^ ^18^ every effort should be made to reduce aerosol emission during dental procedures. The increase in the number of teeth extractions in six of the seven health macro-regions analyzed may be related to this attempt to reduce the aerosol emission, mainly linked to endodontic treatment.^19^ ^,^ ^20^ However, the increase in tooth extractions may also reflect the worsening of oral health conditions, when there is a need for more invasive procedures, or even when “definitive” treatment for pain is sought, within a perspective of dental mutilation and traditional dental care.^3^

The main finding of this study is the reduction in the performance of dental procedures, which is probably not linked to a decrease in the need for treatment or a decrease in the burden of disease among children. This study only assessed curative dental procedures, i.e., procedures performed when there was a need for intervention to treat an already established disease; however, due to the pandemic, dental care in many locations was limited to emergency care.^3^ These results call for reflection on oral health care offered to children, a period of life when healthy habits are often established, whereby Primary Health Care is the ideal field for oral health promotion and prevention actions.^21^

As a strength of this research, it is important to highlight the systematization of the data collected: the same months were analyzed both before and during the pandemic. The results presented can also help monitoring of deciduous teeth curative treatment in the post-pandemic period. Regarding possible limitations, the study used secondary public data, which may be subject to underreporting. Furthermore, it was not possible to estimate the total population with deciduous teeth that could have received care during the periods analyzed, so that no denominator was available to calculate incidence. Due to the nature of the aggregated data, it was not feasible to perform analysis at the individual level, with the aim of assessing causal effect or association with characteristics of the children and/or their family environment. Nor was it possible to compare dental pediatric preventive actions, because this type of procedure does not have the same sub-classification on the information system used for data collection; it is therefore unfeasible to estimate reduction in pediatric dental care in general.

Based on the above, we conclude that there was a reduction in the number of deciduous teeth dental procedures in the period studied, in all health macro-regions that comprise the state of Rio Grande do Sul. These results can inform the planning of oral health care in this population, considering actions related to the promotion of children’s oral health and the re-establishment of favorable oral health conditions.
